# Genome-wide profiling of histone (H3) lysine 4 (K4) tri-methylation (me3) under drought, heat, and combined stresses in switchgrass

**DOI:** 10.1186/s12864-024-10068-w

**Published:** 2024-02-29

**Authors:** Vasudevan Ayyappan, Venkateswara R. Sripathi, Shaojun Xie, Malay C. Saha, Rita Hayford, Desalegn D. Serba, Mayavan Subramani, Jyothi Thimmapuram, Antonette Todd, Venu Kal Kalavacharla

**Affiliations:** 1 Molecular Genetics and Epigenomics Laboratory, Delaware State University, Dover, DE 19901 USA; 2 Center for Molecular Biology, Alabama A&M University, Normal, AL 35762 USA; 3 Bioinformatics Core, Purdue University, West Lafayette, IN 47907 USA; 4 Noble Research Institute, LLC, Ardmore, OK 73401 USA; 5 Center for Bioinformatics and Computational Biology, University of Delaware, Newark, DE 19716 USA; 6 USDA-ARS, U.S. Arid Land Agricultural Research Center, Maricopa, AZ 85138 USA; 7 Center for Integrated Biological and Environmental Research (CIBER), Delaware State University, Dover, DE 19901 USA

**Keywords:** Switchgrass, Drought, Heat, Stress, Chromatin, ChIP-Seq, Peak, Gene, Gene Ontology, Transcription Factors

## Abstract

**Background:**

Switchgrass (Panicum virgatum L.) is a warm-season perennial (C4) grass identified as an important biofuel crop in the United States. It is well adapted to the marginal environment where heat and moisture stresses predominantly affect crop growth. However, the underlying molecular mechanisms associated with heat and drought stress tolerance still need to be fully understood in switchgrass. The methylation of H3K4 is often associated with transcriptional activation of genes, including stress-responsive. Therefore, this study aimed to analyze genome-wide histone H3K4-tri-methylation in switchgrass under heat, drought, and combined stress.

**Results:**

In total, ~ 1.3 million H3K4me3 peaks were identified in this study using SICER. Among them, 7,342; 6,510; and 8,536 peaks responded under drought (DT), drought and heat (DTHT), and heat (HT) stresses, respectively. Most DT and DTHT peaks spanned 0 to + 2000 bases from the transcription start site [TSS]. By comparing differentially marked peaks with RNA-Seq data, we identified peaks associated with genes: 155 DT-responsive peaks with 118 DT-responsive genes, 121 DTHT-responsive peaks with 110 DTHT-responsive genes, and 175 HT-responsive peaks with 136 HT-responsive genes. We have identified various transcription factors involved in DT, DTHT, and HT stresses. Gene Ontology analysis using the AgriGO revealed that most genes belonged to biological processes. Most annotated peaks belonged to metabolite interconversion, RNA metabolism, transporter, protein modifying, defense/immunity, membrane traffic protein, transmembrane signal receptor, and transcriptional regulator protein families. Further, we identified significant peaks associated with TFs, hormones, signaling, fatty acid and carbohydrate metabolism, and secondary metabolites. qRT-PCR analysis revealed the relative expressions of six abiotic stress-responsive genes (transketolase, chromatin remodeling factor-CDH3, fatty-acid desaturase A, transmembrane protein 14C, beta-amylase 1, and integrase-type DNA binding protein genes) that were significantly (*P* < 0.05) marked during drought, heat, and combined stresses by comparing stress-induced against un-stressed and input controls.

**Conclusion:**

Our study provides a comprehensive and reproducible epigenomic analysis of drought, heat, and combined stress responses in switchgrass. Significant enrichment of H3K4me3 peaks downstream of the TSS of protein-coding genes was observed. In addition, the cost-effective experimental design, modified ChIP-Seq approach, and analyses presented here can serve as a prototype for other non-model plant species for conducting stress studies.

**Supplementary Information:**

The online version contains supplementary material available at 10.1186/s12864-024-10068-w.

## Introduction

Switchgrass is cultivated across a vast geographical area, and it is economically important as it produces relatively higher cellulosic biomass with little water and nutrient input for bioenergy production. Growing biomass crops such as switchgrass on marginal lands is sustainable, and it helps in mitigating climate change, as they reduce CO2 emissions over four times more effectively than forest and grassland ecosystems. Since the US Department of Energy (DOE) identified switchgrass as a model biofuel crop, molecular and genomic breeding studies have gained momentum [1]. The ecotype- or genotype-specific genomic/epigenomic variation linked with stress tolerance and cellulosic biomass production can be exploited using biotechnological, and Next Generation Sequencing (NGS) approaches [2]. The polyploid (1C = ~ 1,130 Mb) genome of the lowland ecotype of switchgrass, AP13, that is used in this study is large and repetitive (~ 60%) [2]. Climate models predicted that the United States would be affected by moderate-to-severe drought and excessive heat in the next two decades due to climate change and global warming [3], which may decrease the productivity of agronomically important crops, including C3 and C4 grasses.

Plants are sessile and often are exposed to various abiotic stresses, such as heat and drought, either individually or in combination, adversely affecting plant growth, development, and productivity [4]. Drought is a meteorological condition where the plants receive inadequate rainfall and often show a high evapotranspiration rate and low water-holding capacity around the rhizosphere [4, 5]. Drought can influence plants' molecular, biochemical, and physiological processes, resulting in yield loss [4, 5]. Heat stress is often referred to as a condition when plants are exposed to excessive air temperatures for prolonged periods [6, 7]. Heat stress can influence physiological processes, such as photosynthesis, respiration, transpiration, membrane transport, homeostasis, and osmotic regulation, resulting in plant yield loss [6, 7]. The essential genes, pathways, regulatory networks, underlying molecular mechanisms, and interplay associated with plant drought and heat stress responses are not fully understood. Though drought and heat can independently induce stress-responsive genes and their associated pathways, it is quite possible that plants are exposed to multiple stresses [7], often a combination of drought and heat. When multiple stresses co-occur, plants respond simultaneously by triggering a few transcription factors (TFs), signaling molecules, and pathways. Complex crosstalk or interaction between key, intermediate, and regulatory biomolecules happens when plant stresses co-occur [8].

The concurrent effect of water deficit and high temperature on the growth and yield of crops under drought and heat stresses have been reported [9]. However, we are still trying to understand to what extent the interactive effects of heat and drought would affect the physiological responses of crops. Also, there is no clear evidence to support how crops would recover from heat, drought, and combined stresses. The consequence of drought and heat stress on carbon fluxes and storage was studied in switchgrass [10]. A previous report suggested that drought significantly affects various stages of switchgrass, and yield is dramatically reduced after three consecutive years of drought [11]. The authors further suggested that the early stages of switchgrass growth are more important to ensure biomass yield during subsequent years [11]. The physiological and gene expression variation on drought responses has been studied in switchgrass [12]. The role of small RNAs (microRNAs) has been reported in drought and heat stresses in switchgrass [13]. In another study, deep sequencing identified the regulatory role of miRNAs in drought and salinity stresses in switchgrass [14]. Transcriptome analyses identified over 16 heat-responsive genes in switchgrass [15]. Despite significant progress in classical genetics, molecular breeding, and modern biotechnological approaches, developing drought- and heat-tolerant crops are still challenging because of this constantly changing environment due to anthropogenic factors. Moreover, developing tolerant crops for combined stresses by gene stacking or pyramiding is complex and requires understanding genotype- or species-specific gene expression and regulation.

Chromatin immunoprecipitation-based sequencing (ChIP-Seq) is remarkable due to its high sensitivity and specificity in identifying protein-DNA interactions across the genome and provides high-resolution epigenomes. This technology has proven to be an efficient tool for generating genome-wide histone marks in Arabidopsis [16], rice [17], maize [18], brassica [19], and poplar [20]. The methylation and deacetylation of histone H3 lysine 9 (H3K9) and H3K27 are often associated with gene repression. While the acetylation and methylation of H3K4 and H3K36 are often associated with transcriptional activation in plant stress responses [21]. The positive correlation of H3K4me3 levels with the change in gene expression was first reported in Arabidopsis when subjected to dehydration stress [21]. Also, the genome-wide H3K4me3 modifications associated with genes responsive to drought stress have been reported in rice [22]. Using multiple NGS approaches, it is demonstrated that various epigenetic factors (H3K4me3, DNA methylation, and small RNAs) interact in a coordinated fashion to regulate the expression of heat stress-responsive genes in Arabidopsis [23]. The expression of heat shock proteins (HSPs) was altered in Arabidopsis when subjected to multiple abiotic stresses [24]. However, epigenomic modifications, including H3K4me3 in combined drought and heat stresses, have not been reported in switchgrass. As the transcriptionally active chromatin is marked by H3K4me3 around transcription start sites [25], genome-wide changes in gene expression in response to stress can be studied using ChIP-Seq. Therefore, this study evaluated genome-wide marking of H3K4me3 in switchgrass in response to drought, heat, and combined stresses using ChIP-Seq analysis.

## Materials and methods

### Experimental design and assessment of data quality

We adopted the cost-effective experimental design reported in our previous study on switchgrass [26]. This study used Alamo, AP13 genotype, a lowland ecotype. The selection of the AP13 from the switchgrass cultivar 'Alamo' was initially made at the University of Georgia. Then the genotype was relocated, and clonal copies were maintained at the greenhouse of Noble Research Institute, LLC, Ardmore, OK. The ramets of AP13 were then shifted into the 3-gallon nursery pots in the greenhouse, maintained under optimum conditions for 40 days, and then transported to growth chambers at the Noble Research Institute, LLC. Then the experiment was conducted with three biological replicates in a randomized complete block design starting five days after transfer to the growth chamber.

**Fig. 1:**
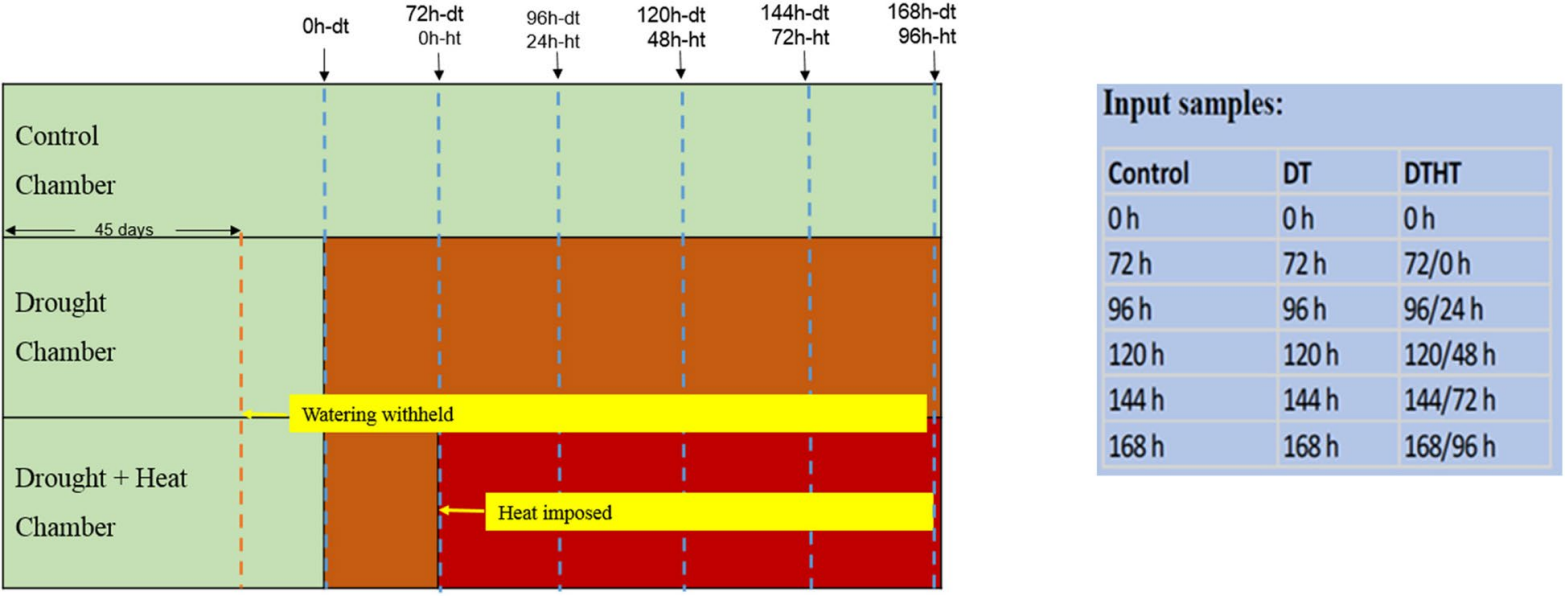
Experimental design of drought and combination of drought and heat treatments in switchgrass. Control chamber: Regular watering (80% FC) and optimum temperature (30°/23 °C day/night temperature); Drought chamber: withhold watering at 45 days after transplanting the ramets and kept at optimum temperature (30°/23 °C day/night temperature); Drought + Heat chamber: imposed heat after 72 h of drought (35°/25 °C day/night temperature); Leaf tissue samples were collected at 0 h-drought (dt), 72 h-dt/0 h-heat (ht), 96 h-dt/24 h-ht, 120 h-dt, 48 h-ht, and 144 h-dt/72 h-ht impositions

We randomly chose six pots for control (C), nine for DT, and nine for combined DT and HT (DT followed by HT) treatments during the transfer. The pots were separated into three treatments, randomly split into three groups, and maintained a minimum of three replicates per treatment. A diagram that shows how the growth chamber was partitioned for DT and HT treatments is depicted (Fig. 1). The control and DT treatments were maintained in a growth chamber. The DT inflicted with HT treatment was kept in another growth chamber, similar brand and model. The replicates for control and treatments were randomly distributed in the chamber (Fig. 1). In addition, for each condition, we maintained a positive/input control PC to compare it against the conditions and time points. The leaf samples were collected around the same time (~ 2:00 PM) and in the same position for all the treatments, with five leaves of the same age from major tillers (that had > 10 fully developed leaves) pooled per replicate. The plant tissues thus collected were flash-frozen using liquid nitrogen (LN2) and later stored at -80 °C for further processing.

### Isolation and immunoprecipitation of chromatin

ChIP assay was carried out as described previously [27] and modified for Switchgrass. First, the finely powdered samples were settled in cold nuclear isolation buffer consisting of 1% formaldehyde with 20 µl of protease inhibitor (Thermo Scientific, Waltham, MA; Product #87786) at room temperature. Next, the sonicated-nuclear lysate chromatin samples were incubated with five µg of rabbit polyclonal antibody raised against a synthetic peptide corresponding to trimethyl-lysine 4 of histone H3 (Active Motif, Carlsbad, CA; 39915) along with 20 µl of Pierce protein A/G magnetic beads (Thermo Scientific, Waltham, MA) and incubated at 4 °C overnight. A negative control sample was processed similarly without adding the antibody. Additionally, input (positive control) samples were maintained with 50 µl of sonicated chromatin in the elution buffer. Then, the antibody-chromatin complex was washed, eluted, and de-cross-linked with 20 µl of 5 M NaCl and placed overnight at 65 °C. Then eluted ChIP DNA was recovered after Proteinase-K digestion, purified by phenol–chloroform extraction followed by ethanol precipitation using glycogen, and re-suspended in 20 µl of TE buffer. Finally, 100 ng of purified DNA generated a ChIP-Seq library for each treatment.

### ChIP-Seq and data analysis

#### Data collection

In total, 63 libraries were generated from C, DT, DTHT, and PC samples and sequenced on Illumina HiSeqTM 2000 (Illumina, San Diego, CA) at the Delaware Biotechnology Institute (DBI, Newark, DE). Among these, 54 (3 conditions × 6 time-points × 3 replicates) libraries were from C, DT, and DTHT, and 9 (3 conditions × 1 positive/input control × 3 replicates) libraries were from PC. The ChIP-Seq data generated in this study is submitted to the GSE (Genomic Spatial Event database) section of the NCBI under bioproject number GSE196295 for the ChIP-Seq experiment.

#### Quality assessment

Read quality was evaluated using FastQC (v 0.11.2) for all samples, and quality trimming was performed with cutadapt (v 1.13) to remove bases with Phred33 score less than 30, and resulting reads of at least 50 bases were retained. The trimmed and filtered reads were mapped against the bowtie2-indexed *Panicum virgatum* reference genome (v4.1) using bowtie2 (v 2.3.1) [28] with default parameters other than the number of mismatches set to 1. The summary statistics, including the number of sequenced, trimmed, and % mapped reads for C, DT, and DTHT immunoprecipitated and PC samples, were given in Table 1.

**Table 1:** Statistical analyses of total sequences obtained, mapped reads, and percentage of aligned reads to the reference genome in control, drought, and combined drought and heat-treated ChIP-seq samples in switchgrass

Samples	Total reads	Mapped Reads	Percentage
C0h	86,411,976	49,272,258	90.26
C120h	81,302,129	64,740,218	89.66
C144	87,135,353	68,116,000	91.33
C168	84,385,408	71,867,468	94.66
C72	90,436,395	72,078,819	89.21
C96	88,686,263	73,355,142	91.83
0PC	67,883,942	55,192,284	92.38
DT0h	85,580,843	68,209,205	94.94
DT72h	84,272,967	69,676,977	92.90
DT96h	54,620,480	45,036,409	91.66
DT120h	84,685,559	69,477,775	92.48
DT144h	86,457,929	69,995,509	91.13
DT168h	85,656,195	69,443,346	91.02
DTPC	89,412,456	71,398,656	89.56
DTHT0h	87,199,026	71,665,691	92.72
DTHT72/0 h	85,970,740	70,377,120	91.93
DTHT96/24 h	85,456,401	68,803,854	91.07
DTHT120/48 h	86,667,852	69,973,683	91.09
DTHT144/72 h	86,009,043	69,683,056	91.15
DTHT168/96 h	84,461,858	69,747,078	92.68
DTHTPC	85,949,561	70,210,770	91.50

The switchgrass samples were treated with drought, and a combination of drought and heat, ChIP DNA was collected, sequenced, and then ChIP-Seq reads were aligned to switchgrass reference genome *P.virgatum* 4.0

#### Peak calling

Histone modification peaks were detected using SICER (Spatial Clustering for Identification of ChIP-Enriched Regions) (Version 1.1) [29] with the parameters as follows – redundancy threshold of 1, window size of 200, gap size of 200, the effective genome size of 0.7 (taken as a fraction of reference genome of Switchgrass), and FDR threshold controlling significance as 0.05 for each sample relative to their control sample. BAM files generated from Bowtie2 were converted to bed files using BEDTools (Version 2.21.0) [30] and used as input for SICER.

#### Identification of stress-responsive peaks

We prepared our experiment in such a way that additional heat treatment is not required when the heat-responsive peaks can be identified bioinformatically by comparing un-stressed (C), DT, DTHT (drought followed by heat), and input controls, especially when we included different collection time-points (0, 72, 96, 120, 144 or 168 h) in our study. Therefore, differential peaks were identified using different replications with a P-value cutoff of 0.01, window size 200, step size 100, gap size 0, and fragment size 300. To identify DT-responsive peaks, we required a region: 1) to be not identified as a differential peak between the drought treatment group and control group at 0 h; and 2) to be identified as a differential peak between the drought treatment group and control group in at least one of the time-points as follows (72, 96, 120, 144 or 168 h). To identify DTHT-responsive peaks, we required a region: 1) to be not identified as a differential peak between the group with a combination of drought and heat treatment and the control group at 0 h; and 2) to be identified as a differential peak between the group with a combination of drought and heat treatment and control group (C) in at least one of the following time-points (72, 96, 120, 144 or 168 h). To identify HT-responsive peaks, we required a region: 1) to be not identified as a differential peak between the group with a combination of drought and heat treatment and drought group at 0 h and 72 h; and 2) to be identified as a differential peak between the group with a combination of drought and heat treatment and control group in at least one of the time-points as follows (96, 120, 144 or 168 h).

#### Functional analysis of stress-responsive peaks

To investigate how DT and DTHT responsive peaks identified in this study correlated with the corresponding responsive genes in RNA-Seq data reported [25] (NCBI GEO accession # GSE174278), mapped files were screened for overlapping regions (with flanking 2 kb regions included) using intersect in BEDTools. Then GO enrichment analysis was conducted using agriGO for the overlapping responsive genes [31]. Overrepresented GO categories of *P. virgatum* genes were found using the PANTHER Classification System Version 13.1 [32] with default settings (including FDR < 0.05). PANTHER Pathways and the PANTHER classes of proteins were also identified.

Further, to determine functional gene enrichment and differentially acetylated gene interaction networks in Switchgrass, we used the genes identified in SICER for functional annotation. To analyze functionally enriched genes in the SICER analyses, we used the Database for Annotation, Visualization, and Integrated Discovery, DAVID [33]. As in the functional annotation charts, the fold enrichment score obtained from DAVID is defined as the percentage of genes in a given genome class. This study considered clusters with Benjamini factor < 0.05 and fold enrichment > 1.3 as significant and non-informative classifications were eliminated [34]. Pathway analysis was performed using MapMan with customized input files generated explicitly for Switchgrass using the Mercator tool to study the gene functions associated with responsive peaks.

The Mercator tool can batch classify sequences (gene/protein) into functional plant categories and make a draft metabolic network that can be used directly in MapMan software [35]. The experiment file has three possible values: 0, 1, and -1. i) "0" means a given gene was not identified as responsive in a particular condition. ii) "1" means the gene was identified as responsive and showed upregulated in at least one of the comparisons in a specific condition. iii) "-1" means the gene was identified as responsive and showed down-regulated in the comparisons. To make the color scale suitable for visualization, "scale" was adjusted to 1 in MapMan.

#### Visualization of data

Plots were generated using Circos [36], color keys were chosen from ColorBrewer (http://colorbrewer2.org) [37], and figure legends were included in the Circos plot by Inkscape (http://inkscape.org). Then ChIP-Seq data were visualized by Integrative Genome Viewer (IGV; http://software.broadinstitute.org/software/igv/).

### RT-PCR and real-time quantitative RT-PCR (qPCR) validation

The RT-PCR and real-time quantitative RT-PCR (qPCR) validations were performed to assess the amplification of DNA/cDNA by using MyCycler thermocycler (Bio-Rad Laboratories, Hercules, CA) and ABI 7500 real-time PCR (Applied Biosystems, Foster City, CA), respectively. Our ChIP-Seq analysis selected six significant peaks associated with DT- and DTHT-responsive genes also reported in Switchgrass [38]. The details of genes and respective primers are provided in Supplemental Table 1. The selected genes' primers were designed using a TaqMan primer design tool for real-time PCR (GenScript USA Inc., Piscataway, NJ). The cDNA (10 ng) extracted from switchgrass leaves was used as a template for 25 μl qPCR reactions in triplicates, in which 10 μM of primer pairs (Forward and Reverse) and 12.5 μl of SYBR Green (Germantown, MD) PCR Master Mix. PCR conditions for qPCR were as follows: 95 °C for 10 min, 40 iterations of 95 °C for 15 s, and 65 °C for 60 s. To normalize the results, cons7 was used as a constitutive control of expression for all tissue samples. The efficiency of primers was tested and analyzed by using the previously reported 2-ΔΔCT method [39], where ΔΔCT = (CT of gene—CT of cons7) tissue to be observed—(CT of Genex—CT of cons7) leaf tissue. The normalized CT values (ΔΔCT) from qPCR analysis were collected and analyzed using Minitab 17, and the expression results were presented as mean ± SE. One-way ANOVA was done on qPCR experiments for multiple comparisons between the mean of samples.

## Results

### Analysis of ChIP-Seq peaks

ChIP-Seq analysis of 63 frozen leaf samples generated 5,361 million reads (300 bp fragments) at 85 million reads per sequenced sample (Table 1). For each condition, we maintained a positive control, namely 0PC, DTPC, DTHTPC. The total number of reads in C, DT, DTHT, and PC were approximately 1.6, 1.4, 1.6, and 0.7 billion reads, respectively. Deconseq [40] analysis revealed that the data collected was of high quality and devoid of contamination. Mapping of quality ChIP-Seq reads to reference genome (v) revealed that about 90% of the reads from each library were aligned and used for downstream analysis (Table 1).

**Fig. 2:**
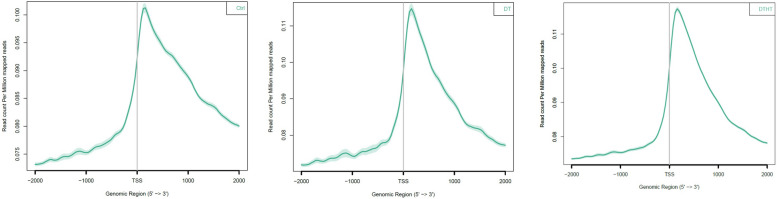
Ngs plot analysis of control, drought, and combination of drought and heat-treated samples in switchgrass. The genomic enrichment of DNA-interacting proteins distanced from the transcriptional start sites (TSSs) has been plotted and they were mapped against per million mapped reads

**Fig. 3:**
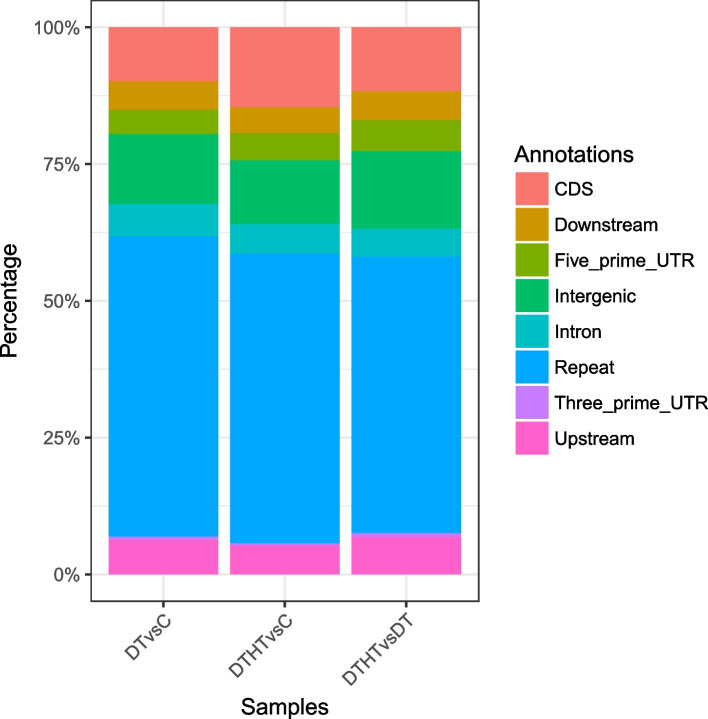
Genome-wide distribution of peaks in drought and combination of drought and heat-treated samples in switchgrass. The location of peaks was identified in both genic and non-genic regions and calculated based on the percentage

Histone modification peaks were detected using the SICER program [29] with a false discovery cutoff of 0.05. In total, 1,374,515 H3K4me3 peaks were identified from all samples (Supplemental Table 2). Among these, DT-H3K4me3 and DTHT-H3K4me3 peaks were 754,409 and 620,106, respectively. On an average, each DT and DTHT sample had 41,900 and 39,481 peaks, respectively (Supplemental Table 3). The median width of a peak in the samples ranged from 1,200 bp to 1,600 bp, and the mean ranged from 1,356 bp to 2,119 bp. On average, the peaks identified on the mapped genome were about 68 Mb per sample. The sites of the enriched peaks on the switchgrass genome are shown (Supplemental Table 2). H3K4me3, an active histone modification that is associated with a transcriptionally active state of chromatin, exhibits conventional and non-conventional patterns, which are generally conserved in most eukaryotes. The canonical H3K4me3 pattern is narrow and associated with actively transcribed promoters and CpG islands. In contrast, the non-canonical H3K4me3 pattern is broad and associated with transcribed gene bodies. In our study, the distribution of enriched H3K4me3 peaks was higher downstream of the transcription start sites (TSS) of transcriptionally active genes (Figs. 2 and 3). While a few peaks (< 50%) were found in genic regions (gene body and flanking 2 kb areas), including CDS, downstream (2 kb from the downstream of the stop codon), 5’-UTR, and upstream (from 2 kb upstream of TSS) elements (Supplemental Table 2; Supplemental Fig. 1).

### Identification of stress-responsive peaks

**Fig. 4:**
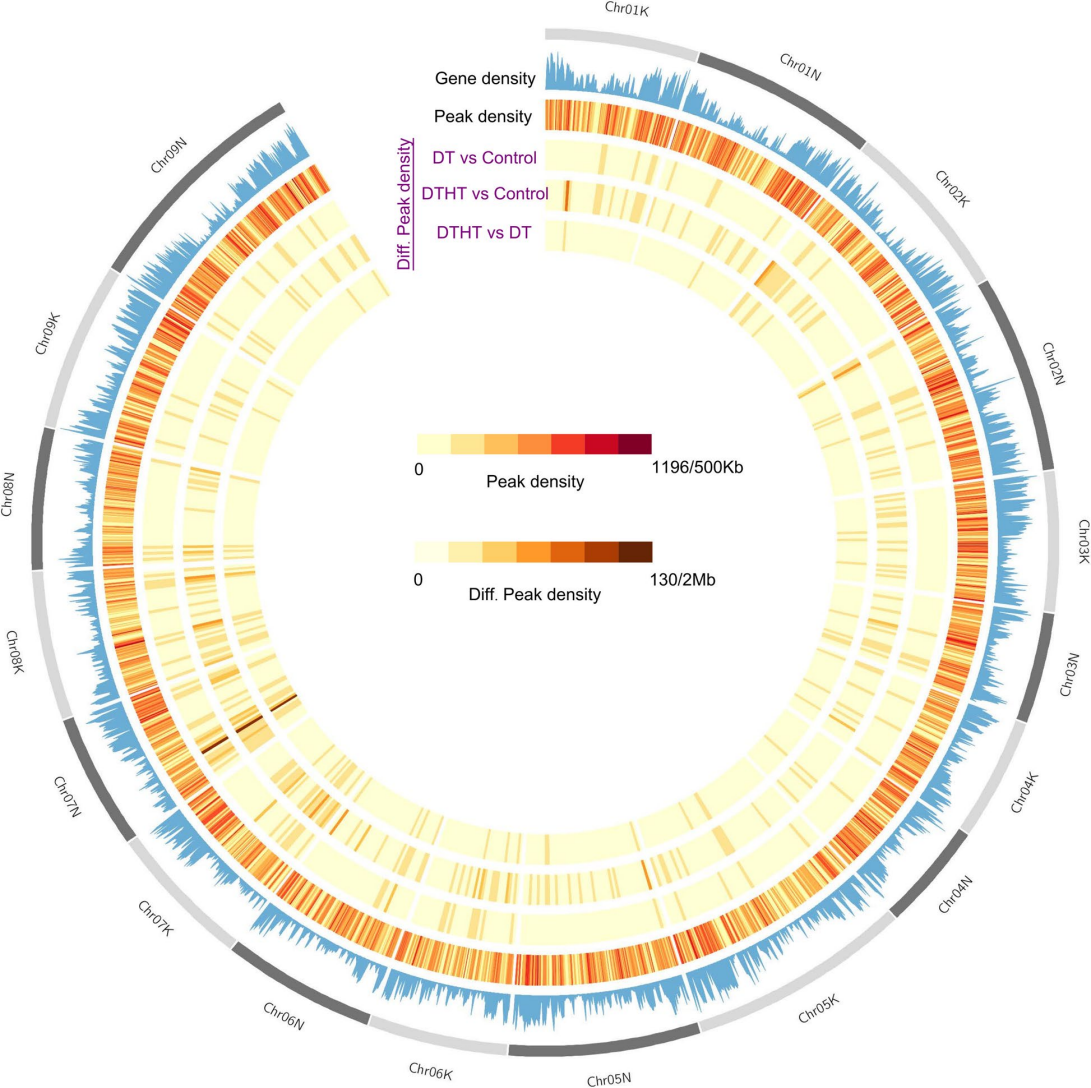
Circos plot analysis of differentially marked H3K4me3 peaks in drought and combined stress of drought and heat in switchgrass. The color indicates density of differentially marked peaks with that of overall genomic peak density

H3K4me3 is a near-universal chromatin modification at the TSS of active genes in eukaryotes and its levels reflect the rate of transcription. Our results indicate that many transcribed genes are not annotated. Reads per million (RPM) of the differential peaks were presented in Supplemental Table 4, and the distribution of stress-responsive peaks based on genomic features was extracted and presented (Fig. 3). The peaks in genic regions were enriched in 5’-UTR and CDS (Fig. 3). The number of DT, DTHT, and HT stress-responsive peaks identified were 7,342; 6,510; and 8,536, respectively (Table 2). We further studied how the DT- and DTHT-responsive peaks correlate with the corresponding candidate genes identified in previously reported RNA-Seq analyses [25]. It is interesting to report that 155 DT-responsive peaks overlapped with 118 DT-responsive genes (Supplemental Table 5). Similarly, 121 DTHT-responsive peaks overlapped with 110 DTHT-responsive genes (Supplemental Table 5). The overlap of the epigenomic peaks and genes could be seen as an interplay between the master regulators of the drought and heat stress-responsive genes. The nearest gene distribution was determined by BEDTools (v2.21.0) [29]; over 6000 H3K4me3 DT and DTHT peaks were spanned between 0 and + 2000 bases from TSS (Supplemental Table 5). Among the peaks identified, ~ 25% of peaks were enriched above 2000 bp from the TSS. The distribution of enriched H3K4me3 levels 2 Kb upstream and downstream of TSS decreased in both treatments gradually. We provided the GO term enrichment results of the genes with the stress-responsive peaks in Supplemental Table 6. We performed a Circos plot analysis to study the binding patterns of H3K4me3 during heat and drought stress in switchgrass. In the circos plot, we presented peak densities of DT and DTHT treatments in the respective chromosomes by comparing them with the genome-wide peak density (Fig. 4). Chr01K has the lowest peak density among them, and Chr07N has the highest peak density.

**Table 2:** Distribution of SICER peaks based on genomic features in switchgrass

	**DT vs. C**		**DTHT vs. C**		**DTHT vs. DT**	
	Number of peaks	% of total peaks	Number of peaks	% of total peaks	Number of peaks	% of total peaks
**Five_prime_UTR**	327	4.45	326	5.01	485	5.68
**CDS**	720	9.81	947	14.55	995	11.66
**Upstream**	465	6.33	345	5.3	590	6.91
**Intron**	435	5.92	352	5.41	437	5.12
**Three_prime_UTR**	45	0.61	27	0.41	56	0.66
**Downstream**	384	5.23	309	4.75	452	5.3
**Repeat**	4,028	54.86	3,446	52.93	4,312	50.52
**Intergenic**	938	12.78	758	11.64	1,209	14.16
	**7342**		**6510**		**8536**	

The number of peaks and percentage associated with each genomic feature among control, DT, and DTHT is presented

### Functional (GO) analysis of stress-responsive peaks

**Fig. 5:**
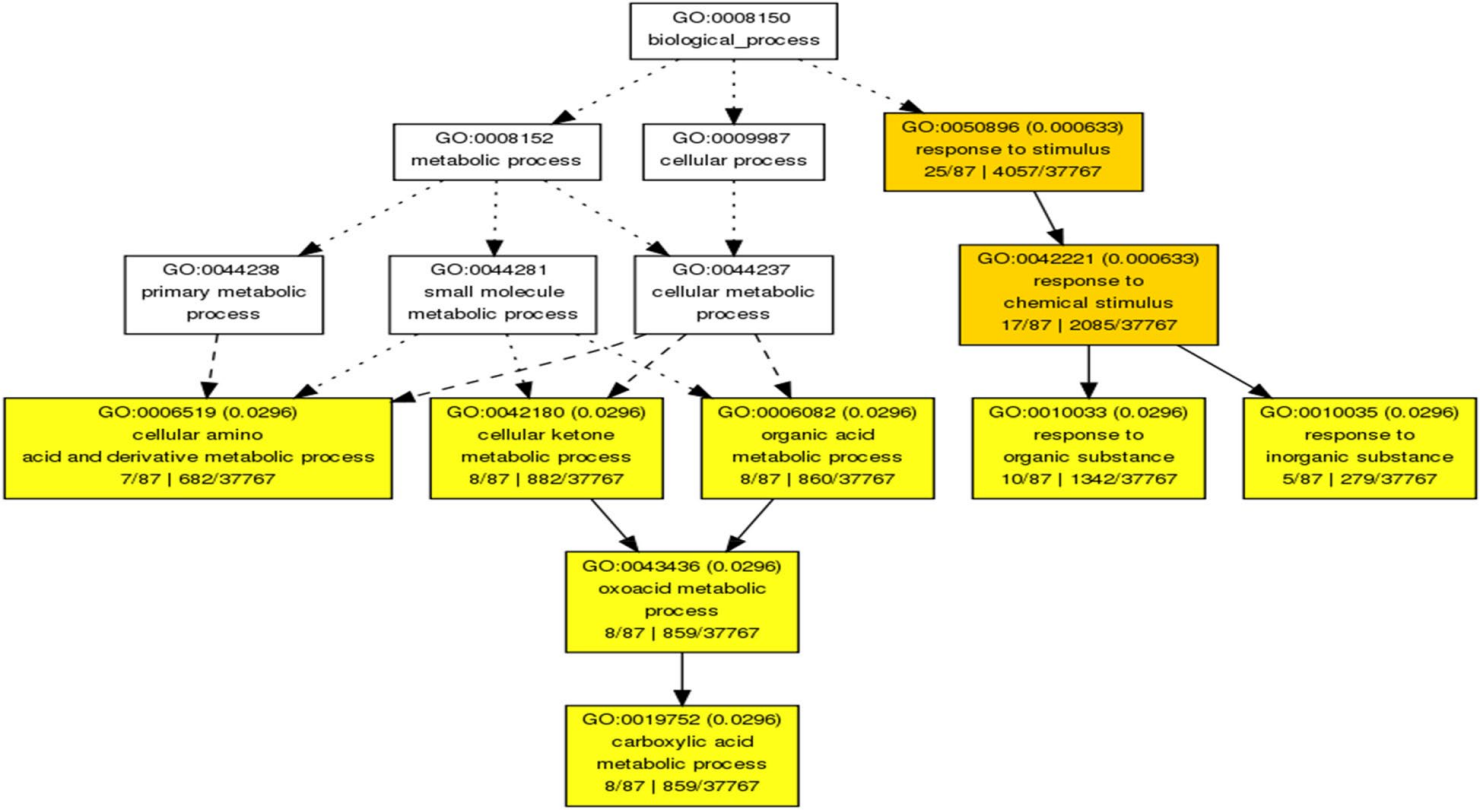
Hierarchical tree graph of overrepresented GO terms in biological, molecular, and cellular process categories generated by AgriGo. Boxes in the graph represent GO terms labeled by their GO ID, term definition and statistical information. The significant term (adjusted *P* < 0.05) are marked with color, while non-significant terms are shown as white boxes. The diagram, the degree of color saturation of a box is positively correlated to the enrichment level of the term. Solid, dashed, and dotted lines represent two, one and zero enriched terms at both ends connected by the line, respectively. The rank direction of the graph is set to from top to bottom

**Fig. 6:**
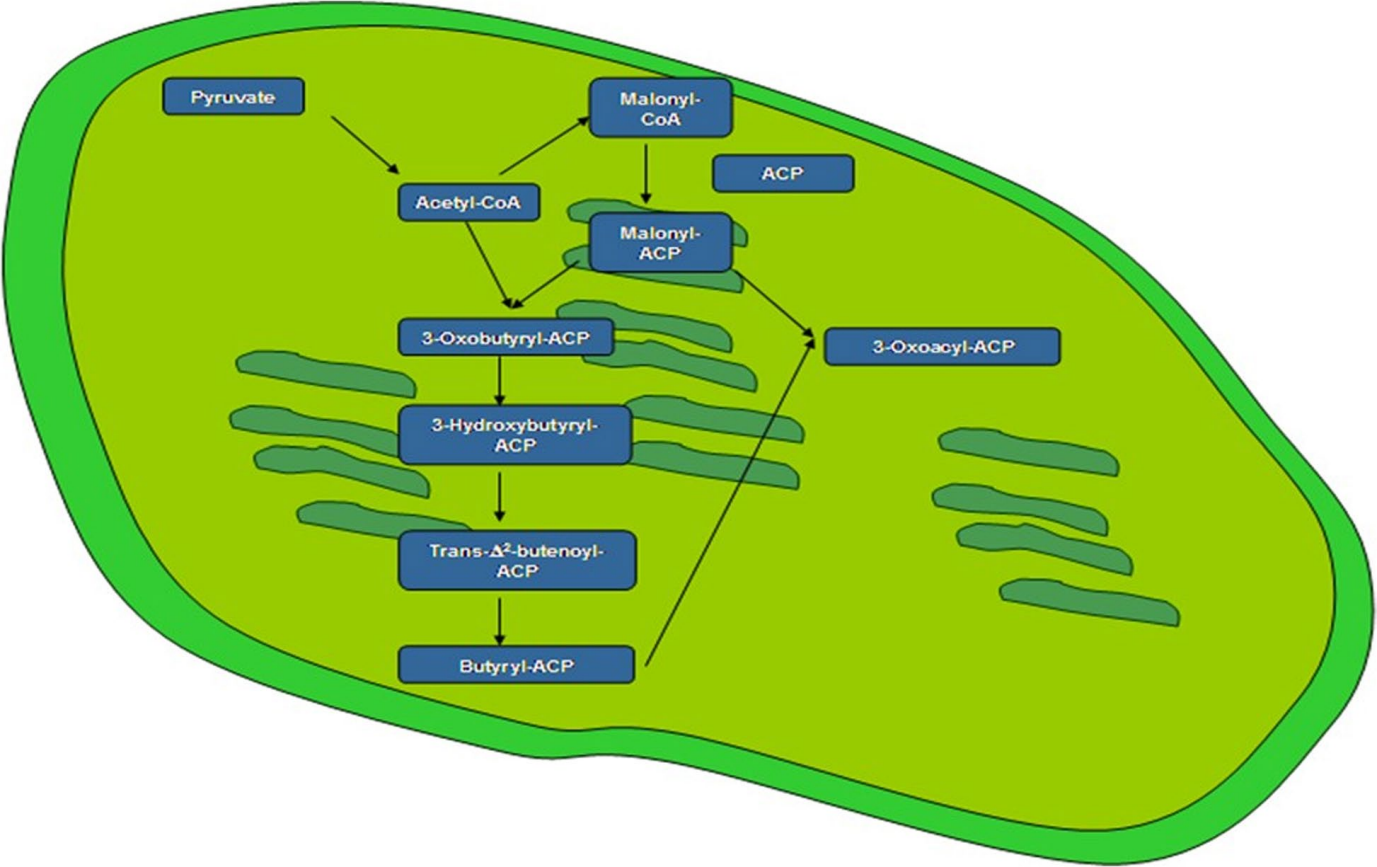
Mapman outline of pathway genes involved in fatty acid metabolism in drought and heat-stressed switchgrass

**Fig. 7:**
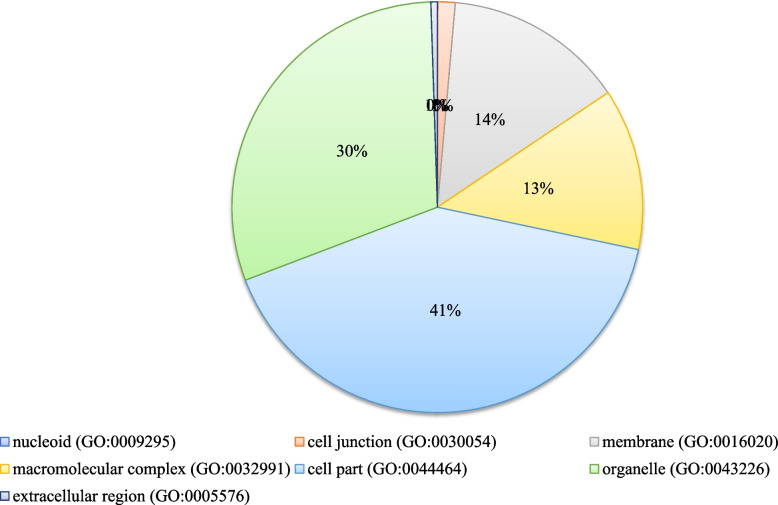
PANTHER classification of differently marked heat and drought-responsive genes in Switchgrass. Generic mapping IDs generated from AgriGo analysis of differentially marked genes were compared with Arabidopsis genome available in online resources

Functional (GO) analysis was performed using agriGO for the overlapping responsive genes (http://bioinfo.cau.edu.cn/agriGO/analysis.php). GO terms for the genes were extracted from “Pvirgatum_450_v4.1.annotation_info.txt”. We identified all three GO categories with significantly over-represented members: Biological Process (45), Molecular Function (28), and Cellular Component (8) when DTHT was compared against DT ChIP-Seq. Similarly, the GO categories were identified between DT and C, and DTHT and C ChIP-Seq samples of switchgrass (Supplemental Table 6). The functional (GO) categories significantly (FDR < 0.05 and p-values < 0.01) enriched in biological processes were: metabolic processes (cellular, primary, nitrogen compound, and macromolecular), cellular processes, and response to stimuli (Fig. 5). Four members of molecular function category that significantly enriched were: transmembrane transporter, transferase, catalytic, and binding activities (Supplemental Table 6). Mapman analysis was performed to study the relationship between heat tolerance and fatty acid metabolism. Our results showed genes involved in fatty acid synthesis were highly marked during the HT and DT stresses (Fig. 6). We performed pathway analysis of the enriched differentially marked genes using the PANTHER database. Most of the differentially marked genes in cellular component category were classified as cell parts (41%), organelle (30%), membrane activity (14%), and macromolecular complex (13%) (Fig. 7).

### Transcription factors (TFs) associated with stress-responsive peaks

This study identified various TFs that were involved in DT, DTHT, and HT stresses. The list of TFs involved in DT treatment was given in Tables 3, 4 and 5. There were five TFs viz., ABA inducible bHLH type (AtAIB), MADS-box protein Soc 1, growth-regulating factor, chaperone-protein dnaj-related, and cleavage and polyadenylation specificity factor 100. The TFs that responded during HT stress were KUA1, MADS-box protein Soc 1, CW-type Zinc finger, and NAC-domain containing protein 86. The TFs that were enriched during DTHT stress were auxin-response factor 16 (ARF 16), ABA-inducible bHLH types A1B, BR1-EMS suppressor (BES), chromatin remodeling factor CHD3, LEAFY (LFY), and myeloblastosis (MYB) (Tables 3, 4 and 5). This study also revealed various genes that responded to the stimulus. The commonly identified gene in DT and DTHT was L-type lectin domain-containing receptor-like protein kinase IV.1 (LFCRK41) (Tables 6, 7 and 8). The other stimulus-responsive genes identified belonged to chloroplastic exonuclease V, cysteine-rich receptor-like kinase 8 (CRK8), and 17.4 kDa class III heat-shock proteins, HSPs. We also identified various transporter genes that were marked in DT and DTHT treatments. The important transporter genes differentially marked in DT and DTHT stresses were ABC transporter C family member 3 and calcium-transporting ATPase-8 (Tables 6, 7 and 8).

**Table 3:** List of transcription factors in response to drought stress compared to control (DT vs. Ctrl) in switchgrass

S.No	Description	Gene ID
1	ABA Inducible bHLH-Type (AtAIB)	Pavir.5NG443900
2	MADS-box protein Soc 1	Pavir.5KG302900
3	Growth regulating factor (GRF)	Pavir.7KG075300
4	Chaperone-protein dnaj-related	Pavir.6KG359300
5	Cleavage & polyadenylation specificity factor 100	Pavir.J263400

**Table 4:** List of drought and heat-responsive transcription factors compared with control (DTHT vs. Ctrl) in switchgrass

S.No	Description	Gene ID
1	KUA1	Pavir.2KG178200
2	MADS-box protein Soc1	Pavir.5KG302900
3	CW type Zinc finger	Pavir.3NG109300
4	NAC domain containing protein 86	Pavir.5KG170000

**Table 5:** List of drought and heat-responsive transcription factors compared with drought stress (DTHT vs. DT) in switchgrass

S.No	Description	Gene ID
1	Auxin response factor 16 (ARF16)	Pavir**.**9KG184300
2	ABA-inducible bHLH type A1B	Pavir.5NG443900
3	BRI 1-EMS suppressor (BES)	Pavir.3KG414500
4	Chromatin remodeling factor (CHD3)	Pavir.5KG674400
5	LFY	Pavir.6KG378900
6	MYB	Pavir.7NG354300

**Table 6:** List of drought stimulus genes in response to drought treatment compared to control (DT vs. Ctrl) in switchgrass

S.No	Description	Gene ID
1	L-Type lectin domain containing receptor kinase IV.1 (LFCRK41)	Pavir.7NG164900
2	Cysteine-rich receptor like protein kinase 8 (CRK8)	Pavir.8NG316700

**Table 7:** List of drought and heat stimulus genes in response to combined drought and heat treatment (DTHT vs. Ctrl) when compared to control in switchgrass

S.No	Description	Gene ID
1	L-Type lectin domain containing receptor kinase IV.1 (LFCRK41)	Pavir.7NG164900
2	Chloroplastic Exonuclease V	Pavir.4NG327400

**Table 8:** List of drought and heat stimulus genes in response to combined drought and heat treatment when compared to drought (DTHT vs. DT) in switchgrass

S.No	Description	Gene ID
1	L-Type lectin domain containing receptor kinase IV.1 (LFCRK41)	Pavir.7NG164900
2	Chloroplastic Exonuclease V	Pavir.4NG327400
3	17.4 kDa class III heat-shock protein	Pavir.2KG169300

### Validation of ChIP-Seq results

**Fig. 8:**
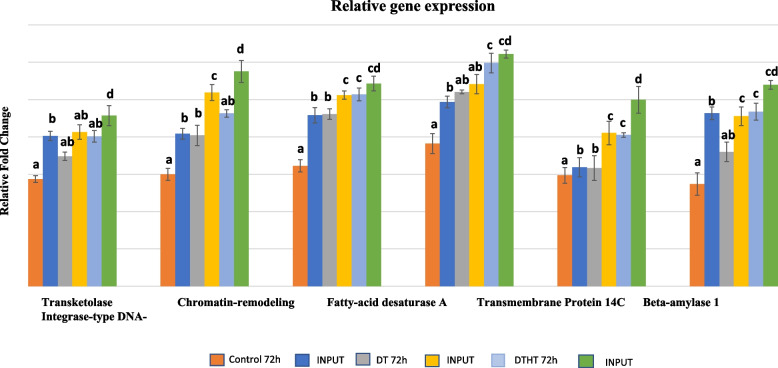
Differential expression of genes in control, drought and combination of drought and heat-treated switchgrass samples in H3K4me3 modification using Quantitative-Real-Time PCR (qPCR) analysis. The normalized CT values (ΔΔCT) from qPCR analysis were collected and analyzed by using Minitab 17, and the expression results were presented as mean ± SE (a—f). One-way ANOVA was performed on qPCR experiments for multiple comparisons between the mean of samples

**Fig. 9:**
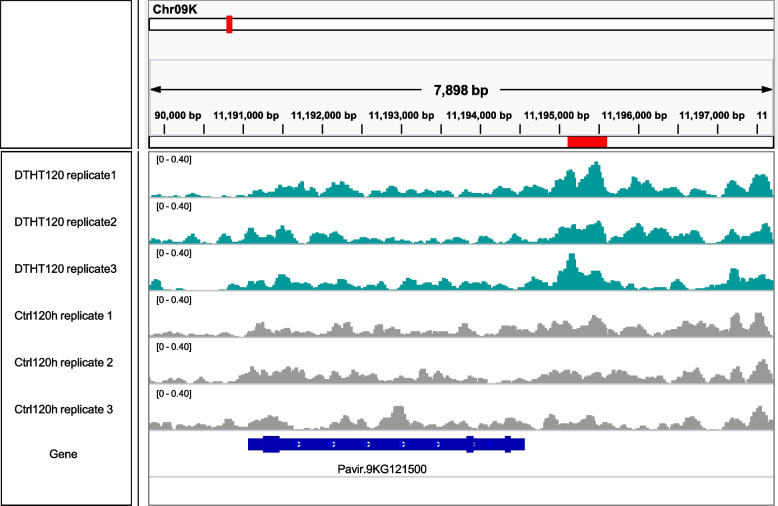
Comparative visualization of a representative region of Auxin Response Factor 16 gene on chromosome 9 K of control, drought and combination of drought and heat-treated samples in switchgrass. Seventy-two hour-treated drought and drought and heat samples (blue) were compared with INPUT (positive control in red) as background using Integrative Genomics Viewer (IGV)

To validate our results from ChIP-Seq analysis, ChIP-qPCR was conducted using triplicates for control and stressed leaf samples. Our comprehensive ChIP-Seq analysis revealed 162, 185, and 125 genes correlated with peaks between DT vs. C, DTHT vs. C, and DTHT vs. DT, respectively, for H3K4me3 modification. Among these, six essential genes (Supplemental Table 5) involved in abiotic stress tolerance have been selected to validate the expression level by qPCR. The relative expressions of all six genes indicated significant (*P* < 0.05) marks under drought and heat stresses compared to combined stress treatment. Further, for all the treatments, the expression levels were higher for transmembrane protein 14C, followed by fatty acid desaturase A, and chromatin-remodeling factor CHD3 (Fig. 8). The expression of the above-validated genes by ChIP-qPCR correlated with our ChIP-Seq analysis. Furthermore, the distribution of histone H3K4me3 peaks in the Auxin responsive factor 16 gene on chromosome 09 K was visualized using IGV genome browser (Fig. 9).

## Discussion

### Analysis of ChIP-Seq peaks

Histone H3 lysine 4 trimethylation (H3K4me3) has been used as an active mark to identify histone modifications associated with drought-responsive genes in Arabidopsis [21, 41]. A substantial increase in H3K4me3 activity in response to heat stress has been reported in Arabidopsis [21]. Similarly, we utilized H3K4me3 mark to detect transcriptionally active genes that respond to drought (DT), heat (HT), and combined drought and heat (DTHT) stresses. This is the first ChIP-Seq report on DT, HT, and DTHT stresses in switchgrass.

This study presents a modified ChIP-Seq pipeline for preprocessing and analyzing H3K4me3 data in AP13, a lowland switchgrass ecotype. This helps us to investigate the role of unfathomed epigenetic mechanisms in switchgrass that are likely linked with habitat preference. Several lines of evidence evaluated the efficacy of our pipeline: 1) ~ 89.21 – 94.66% of reads were aligned with the reference genome, which is comparable to the results obtained from other contemporary studies in plants, for example, Arabidopsis (95%) [42] brassica (93.0–98.0%) [43], sorghum (97.0%) [44], rice (90.0%–99.0%) [45], and maize (85.0%–92.0%) [46]. 2) The number of uniquely mapped H3K4me3 peaks (7,342, 6,510, and 8,536 peaks were responded to DT, DTHT, and HT, respectively) identified in our study were smaller when compared with those in Arabidopsis [42, 47], brassica [43, 48], rice [45], sorghum [44, 49], and maize [46, 49], yet the genome size of switchgrass (1129.9 Mb) is larger than Arabidopsis (~ 140 Mb), brassica (~ 920 Mb), rice (~ 430 Mb), and sorghum (~ 730 Mb), which may be due to lack of high-quality annotations in switchgrass. 3) We found diverse H3K4me3 distribution patterns in switchgrass, predominantly enriched in the genic regions and TSS, consistent with previous studies in other organisms [42–53]. 4) Our ChIP-Seq analysis was compared with RNA-seq data to identify the relationship between actively transcribed genes and genes altered by H3K4me3. However, the mean expression level of unmarked genes is lower than H3K4me3 marked genes. 5) Gene annotation and ontology analysis of H3K4me3 showed the enrichment of genes in cellular and metabolic processes and signaling. In addition, the H3K4me3 distribution patterns and functional annotations found in this study were comparable with previous reports in other plant species, indicating the high dependability of our ChIP-Seq data.

### Identification and Functional analyses of stress-responsive peaks

After alignment, we annotated the peaks for genic and non-genic regions. We identified that the peaks in the genic regions were mainly enriched in 5'-UTR and CDS regions in switchgrass. Our study is in accordance with a recent report that showed the enrichment of peaks in the SUMO-associated genes, primarily around TSS, promoter-proximal region, and putative 5' UTR regions in rice [54]. In another report, enriched H3K4me3 histone modification correlated with the transcription of 3' upstream and TSS regions [41]. Most of the H3K4me3 modifications have been identified at the 5'-ends under drought treatment in Arabidopsis [41]. In addition, an increase in H3K4me3 trimethylation in the promoter region has been reported in Arabidopsis [21].

Our functional analyses showed the enrichment of H3K4me3 around the TSS and 5’end of the transcriptionally active genes, which is consistent with the distribution of histone modifications in other plants (Arabidopsis [55], rice [44, 51], sorghum [49], apple, [56], soybean [57], eucalyptus [58]), implying a conserved regulatory role of H3K4me3. In addition, we assigned the functions associated with the genes marked by H3K4me3. The H3K4me3 marked peaks that overlapped with annotated genes mostly belonged to molecular regulation, physiological processes, and energy metabolism, indicating the involvement of H3K4me3 in controlling housekeeping genes [59] and stress-induced signaling pathways [60, 61]. The H3K4me3 peaks corresponded with the annotated genes and were more likely to be activated, including the highly expressed genes in response to abiotic stresses, for example, 17.4 kDa class III HSP, chloroplast exonuclease V, ABA Inducible bHLH-Type (AtAIB), MADS-box protein Soc 1, NAC domain-containing protein 86, Auxin response factor 16 (ARF16), and Chromatin remodeling factor (CHD3). Most of these genes linked with stress-related processes altered by H3K4me3 have also been reported in other plant species [61–64]. These findings suggest that H3K4me3 probably modulates active transcription in switchgrass but with a distinct pattern of genomic distributions and regulatory roles. However, in contrast to the previous findings [65–67], H3K4me3 marks were widely distributed in switchgrass. We identified 22% (1950 out of 8536) of all the peaks were enriched around TSS and 34% (3015 out of 8536) in genic regions in switchgrass. This is compatible with recent findings in Arabidopsis that the H3K4me3 mark was significant in TSS and genic regions [60, 65, 68, 69], indicating that domains might be conserved in organisms [45, 70, 71]. Species-specific H3K4me3 distribution patterns were reported in plants, while some genomes were highly methylated, and a few were sparsely methylated [72–74]. The epigenetic mechanisms often act in a coordinated fashion to influence plant functions. Our results indicate that most H3K4me3 distribution and regulation were conserved, while a few are phylogenetically unique.

Our results indicated that most of the H3K4me3 peaks responded to DT, DTHT, and HT were enriched within 2000 bp from the TSS of protein-coding genes. A previous study reported over 40% of the HSFA1b-bound genes spanned around 250 bp from the TSS under heat stress in Arabidopsis [75]. In another study, the enrichment of OSABF1V has been found close to 200 bp of the TSS site in response to drought stress in rice [76]. The H3K4me3 mark is prevalent in actively transcribing genes in Arabidopsis [77]. Our study identified 155, 121, and 175 genes marked by H3K4me3 modification in response to DT, DTHT, and HT, respectively. A previous rice study showed a direct correlation between the enrichment of the H3K4me3 and 423 drought stress-responsive genes [22].

RNA-dependent RNA polymerase (RDR) family protein is involved in various abiotic stresses. Our study identified the multiple copies of the RDR family protein on chromosome 07. Among them, RDR6 is the most responsive to various stresses. The ChIP-Seq analysis of 15-day-old seedlings identified H3K4me3 enrichment in four drought-responsive genes including RDR6 under the dehydration stress in plants [78]. The positive correlation between H3K4me3 abundance and RDR6 in dehydration stress has been reported in 4-week-old rosette Arabidopsis leaves [79]. Histone-mediated regulation of the RDR6 gene under high temperatures has been observed in Arabidopsis [80]. A recent study reported an essential role for sRDR6 in PEG-induced drought stress in *Saccharum spontaneum* [81]. In another study, HvRDR2 has been significantly induced in response to heat stress in barley [82].

Gene Ontology analysis revealed various GO categories involved in biological, molecular, and cellular processes of switchgrass. Most biological processes identified here belonged to transmembrane transporter, transferase, catalytic, and binding activities. Similar to a previous drought stress study in rice [83], we found a significant number of drought-responsive genes that belong to transmembrane/transporter activity, plant hormones, and carbohydrate metabolism categories in switchgrass. We found ten genes commonly expressed or marked between two datasets by comparing ChIP-Seq and RNA-Seq analyses of DT, DTHT, and HT stresses. Among these, the essential genes that triggered in response to abiotic and biotic stresses with known functions were: 1) Aldolase-type TIM barrel family protein, also known as pyridoxal 5'-phosphate synthase pdxS subunit (pdxS and pdx1), which is involved in protein binding and cofactor biosynthesis that plays a role in protecting cellular membranes from lipid peroxidation [84]; 2) Condensin, subunit H, is an essential gene required for chromosome stability, condensation, and segregation [85]; 3) NB-ARC domain-containing or LRR containing proteins that are known to be involved in resistance to plant stresses [86]; 4) Pectin acetylesterase family protein or Notum-related proteins that play a role in structural stability, binding, and catalytic activity [87]; 5) Putative Mg-protoporphyrin IX chelatase subunit CHLH that is involved in chlorophyll biosynthesis [88]; 6) Receptor lectin kinases that are involved in plant development and stresses [89]; and 7) rRNA N-glycosylase/N-glycosidase enzymes that are induced during abiotic stresses [90] (Table 9).

**Table 9:** List of ten commonly identified genes between RNA-Seq and ChIP-Seq datasets in response to combined stresses (DT and DTHT) compared with control (Ctrl) in switchgrass

S.No	RNA-Seq DT Vs ChIP-Seq	Annotated Function	Gene Location
1	Pavir.1KG144100.v4.1	Unknown	Chr01K:22691501–22694137
2	Pavir.7KG075300.v4.1	Putative Mg-protoporphyrin IX chelatase subunit CHLH	Chr09N: 82556622–82558235
3	Pavir.8NG086600.v4.1	rRNA N-glycosylase / rRNA N-glycosidase	Chr08N: 11955020–11971632
4	Pavir.5KG302800.v4.1	Unknown	Chr05K: 54419685–54421587
5	Pavir.4KG101000.v4.1	Unknown	Chr04K: 9854490–9864065
6	Pavir.2KG178200.v4.1	Receptor lectin kinase	Chr02K: 24333006–24334305
7	Pavir.5KG302900.v4.1	Chromosome condensation complex Condensin, subunit H	Chr05K: 54421971–54427469
8	Pavir.J191800.v4.1	Pectinacetylesterase family protein or Notum-related protein	Scaffold_1825: 10616–14690
9	Pavir.8KG189000.v4.1	NB-ARC domain-containing disease resistance protein or LRR containing protein	Chr08K: 31363953–31374075
10	Pavir.2NG003200.v4.1	Aldolase-type TIM barrel family protein or pyridoxal 5'-phosphate synthase pdxS subunit (pdxS, pdx1)	Chr02N: 356596–359236

### Transcription factors (TFs) associated with stress-responsive peaks

TFs regulate the expression of gene/s or gene sets or networks that often respond to various abiotic and biotic stimuli. For example, MADS-Box proteins are crucial in triggering gene regulatory webs that impart drought stress tolerance in plants [91]. This study identified the MADS-Box protein Soc 1, a highly conserved DNA binding domain-containing transcriptional factor in both drought and combined (drought and heat) stresses. Often MADS-domain proteins interact with DNA binding sites at the CArG box in the promoter region to initiate transcription by recruiting co-factors and chromatin remodeling proteins [92].

The activation mark H3K4me3 recruits TFs, co-factors, and CRFs to modulate gene expression. Another TF, bHLH (basic helix-loop-helix), was found to be a positive regulator for root development and drought tolerance in maize [93], peanut [94], and Arabidopsis [95]. We found two bHLH TFs, ABA inducible bHLH-type (AtAIB) and bHLH DNA binding family proteins that were differentially marked by H3K4me3 under drought and combined stresses. In a previous report, AtAIB positively regulated the phytohormone ABA, which activates a significant set of genes involved in signaling when subjected to drought stress in Arabidopsis [96, 97]. We also identified the MYB, NAC, LFY, and zinc finger families of TFs that play a critical role in imparting abiotic stress tolerance. These families of TFs are not direct components of the ABA-induced signaling pathway. However, the direct correlation of ABA sensitivity with the MYB activity resulted in ABA-induced stomatal closure and increased drought tolerance in plants [98].

In rice, OsbZIP23 is identified as a key regulator in ABA signaling and drought resistance [99]. In a recent study, transcription factor OsbZIP23 has been reported to regulate the expression of dehydrin genes directly and positively and H3K4me3 modification levels under drought stress. The use of dehydrin gene as a drought-sensitive marker in investigating gene regulation at both the transcriptional and epigenetic levels has been reported [100]. Another study in rice reported that wide grain 7, a cysteine-tryptophan (CW) domain-containing transcriptional activator may increase the expression of grain size gene (OsMADS1), by binding directly to its promoter, thus increasing the histone H3K4me3 enrichment, and ultimately resulting in increased grain width [101].

The critical role of DNAJ proteins in enhancing drought tolerance and resistance to *Pseudomonas solanacearum* in transgenic tobacco has been reported [102]. In Arabidopsis, the interaction of chaperone DNAJ-like zinc finger with HSP70 in protein homeostasis under heat stress has been reported [103]. In a recent study in rice, the elevated expression levels of DNAJ-proteins at 3 h and 6 h time-points under drought stress has been reported [104]. The NtDnaJ1 gene overexpression in *A. thaliana* exhibited higher DNAJ protein ortholog levels and tolerance to drought stress [105]. The abnormal recruitment of DNAJ1-mediated transcription activation and its role in crosstalk between other epigenetic factors in plants, yeast, and mammals has been reported [106]. The SET- and RING-associated (SRA) domain of SUVH1/SUVH3 that binds all three contexts (CG, CHG, and CHH) of methylated DNA with a preference towards the CHH context was reported in Arabidopsis [106]. Further, these methylated regions recruit DNAJ domain-containing proteins to promote the expression of adjacent genes [106]. Similarly, in rice, when subjected to salt stress, the SRA domain of SUVH7 binds to CHH methylated regions (MITE transposable elements) within the promoter of a Na + /K + transporter (HKT15) to repress the gene expression [107]. While SUVH7 recruits a BAG4 chaperone or an MYB106 TF to promote the activation of genes in the vicinity of HKT15 transporters. The SRA domain of SUVH homologs has a role in evading transposon- or methylation-mediated gene silencing, thus activating the neighboring genes by recruiting chaperone regulators or TFs. The DNAJ-related chaperones enriched in drought, heat, and combined stresses in this study may have similar roles in promoting gene expression.

The auxin response factors (ARFs) are primarily essential for plant growth and development. Also, several reports corroborated the role of ARFs in tolerance to diverse abiotic stresses [108–110]. The extent of changes in auxin distribution and signaling has been investigated in drought stress in tomatoes [108]. They identified many ARFs that potentially mediate the auxin-responsive genes in drought and heat stresses. Similarly, we identified multiple copies of auxin response factor 16 under drought, heat, and combined stresses in switchgrass.

### Stress-induced genes associated with stress-responsive peaks

We identified a plasma membrane protein, receptor-like protein kinase (RLK), that triggers Reactive Oxygen Species (ROS) signaling in abiotic stresses. The cysteine-rich protein kinase (CRK) receptor identified here may act as a sensor to recognize environmental stimuli, and its role in ROS signaling has been suggested in overcoming stress responses. In an earlier study, the overexpression of subfamily CRK45 showed increased tolerance against drought in Arabidopsis 110]. Our study revealed H3K4me3-mediated activation of CRK8 under drought stress, as identified in Arabidopsis [109]. Also, histone-mediated activation was seen in proteins that responded to stimuli, such as LECRK41 protein kinase, chloroplastic exonuclease V, and 17.4 kDa class III HSP.

### Transporters associated with stress-responsive peaks

ATP-binding cassette (ABC) transporter plays a vital role in transporting abscisic acid (ABA) to guard cells to prevent water loss during drought stress [111]. In Arabidopsis, the ABCG40 transporter is localized in guard cells, and it is critical for the proper plant response to ABA. Furthermore, the functional role of the ABCG40 transporter is implicated in importing ABA to stomatal cells and drought stress [112]. In Medicago, the overexpression of the Chr6g0452171 gene that encodes ABC transporter B family protein enriched the histone mark under phosphorus deficiency (PD) and their possible role in PD-induced root system architecture (RSA) remodeling has been reported [113].

Also, the ABC transporter B family proteins are involved in auxin transport and iron homeostasis under PD in Arabidopsis [114]. Similarly, H3K4me3 marked over ten ABC transporter C family member genes in our study in response to drought, heat, and combined stresses, suggesting a functional role in RSA remodeling by modulating ion homeostasis. In poplar, the possibility of a change in phosphorylation is one of the most critical regulatory ways to modulate stress responses has been reported [115]. Also, they identified amino acid transporter (AVT6A-like) as the most interconnected gene with protein phosphatase 4 mediated phosphorylation when subjected to drought stress [115]. Similarly, we found enriched AVT6A-like transporter levels during drought, heat, and combined stresses in switchgrass.

## Conclusion and future perspectives

This is the first genome-wide study investigating heat, drought, and combined stress in switchgrass. We observed an enrichment of H3K4me3 peaks downstream of the TSS of protein-coding genes. Circos plot indicates the peak density of DT and DTHT treatments across the respective chromosomes relative to a genome-wide peak density. GO categories showed significant enrichment primarily in four functional categories: transmembrane transporter, transferase, catalytic, and binding activities. Mapman analysis revealed that fatty acid synthesis genes were highly marked in response to DT and HT stresses. The PANTHER analysis revealed that many genes can be classified into cellular components, organelles, membrane activity, and macromolecular complexes. During combined drought and heat stresses, TFs auxin-response factor 16 (ARF 16), ABA-inducible bHLH type A1B, BR1-EMS suppressor (BES), chromatin remodeling factor CHD3, LEAFY (LFY), and myeloblastosis (MYB) were significantly enriched. ChIP-qPCR analysis showed that transmembrane protein 14C had the highest levels of expression, followed by fatty acid desaturase A, and chromatin-remodeling factor CHD3. Only a few studies are available investigating how combined heat and drought stress affect plant histone modifications. Therefore, results from this study may be helpful in understanding the epigenome regulation in plants exposed to the combination of abiotic stresses.

### Supplementary Information

**Additional file 1: Supplemental Table 1.** List of primers selected for real-time quantitative PCR (q-RT-PCR) from ChIP-Seq.

**Additional file 2: Supplemental Table 2.** ChIP-Seq analysis of differentially marked histone H3K4me3 peaks in response to DT and DTHT.

**Additional file 3: Supplemental Table 3.** Average number of H3K4me3 peaks in DT and DTHT-treated samples.

**Additional file 4: Supplemental Table 4.** Reads per million (RPM) of the differential peaks identified between DT vs Ctrl, DTHT vs Ctrl, and DTHT vs DT.

**Additional file 5: Supplemental Table 5.** DT and DTHT-responsive peaks correlated with corresponding protein coding genes identified by RNA-Seq analysis.

**Additional file 6: Supplemental Table 6.** AgriGO analysis of genes associated with differential peaks involved in biological, molecular, and cellular functions in response to DT and DTHT.

**Additional file 7: Supplemental Table 7.** List of DT responsive genes overlapping DT responsive peaks (for MapMan visualization).

**Additional file 8: Supplemental Table 8.** List of DTHT responsive genes overlapping DTHT responsive peaks (for MapMan visualization).

**Additional file 9: Supplemental Table 9. **List of HT responsive genes overlapping HT responsive peaks (for MapMan visualization).

**Additional file 10: Supplemental Figure 1.** Distribution of stress-responsive peaks based on genomic features.

## Data Availability

The datasets generated and/or analyzed during the current study are available in the GSE (Genomic Spatial Event database) repository, GSE196295 [https://www.ncbi.nlm.nih.gov/geo/query/acc.cgi?acc=GSE196295].

## Abbreviations

DT: Drought
HT: Heat
DTHT: Drought and heat stress
GO: Gene Ontology
IGV: Integrative Genomics Viewer
qRT-PCR: Quantitative Real Time PCR
RPM: Reads Per Million
