# Supplementary material for: Genome-wide profiling of histone (H3) lysine 4 (K4) tri-methylation (me3) under drought, heat, and combined stresses in switchgrass
Source: BMC Genomics. 2024 Feb 29;25:223. doi: 10.1186/s12864-024-10068-w (PMC10903042; doi:10.1186/s12864-024-10068-w)
Supplement: Supplementary file 7 — Additional file 7: Supplemental Table 7. List of DT responsive genes overlapping DT responsive peaks (for MapMan visualization). [file 12864_2024_10068_MOESM7_ESM.pdf]

Supplemental Table 7. List of DT responsive genes overlapping DT responsive peaks (for MapMan visualization)

Gene ID	DTvsC Possible Values
Pavir.J263400.v4.1	1
Pavir.8KG001600.v4.1	1
Pavir.9NG431400.v4.1	-1
Pavir.5NG381400.v4.1	1
Pavir.6KG148100.v4.1	-1
Pavir.2NG068500.v4.1	-1
Pavir.2NG009500.v4.1	-1
Pavir.1NG120500.v4.1	-1
Pavir.3KG332900.v4.1	1
Pavir.5KG646800.v4.1	1
Pavir.7NG362400.v4.1	-1
Pavir.7NG070800.v4.1	-1
Pavir.4KG048500.v4.1	1
Pavir.3NG175400.v4.1	1
Pavir.4KG402100.v4.1	1
Pavir.5NG443900.v4.1	1
Pavir.7KG346000.v4.1	1
Pavir.9NG265200.v4.1	1
Pavir.7NG086200.v4.1	1
Pavir.6KG113300.v4.1	1
Pavir.8NG001000.v4.1	-1
Pavir.1KG490600.v4.1	-1
Pavir.8KG219200.v4.1	1
Pavir.7KG140700.v4.1	1
Pavir.1KG144100.v4.1	1
Pavir.3KG518600.v4.1	1
Pavir.9NG471700.v4.1	1
Pavir.1KG234200.v4.1	1
Pavir.1KG525800.v4.1	-1
Pavir.6KG315300.v4.1	-1
Pavir.1NG173100.v4.1	-1
Pavir.3KG066800.v4.1	1
Pavir.9NG855200.v4.1	-1
Pavir.6KG113600.v4.1	1
Pavir.5KG482900.v4.1	-1
Pavir.7NG180700.v4.1	1
Pavir.7NG021500.v4.1	-1
Pavir.9KG518000.v4.1	1
Pavir.6NG371600.v4.1	1
Pavir.1NG520900.v4.1	1
Pavir.8KG173000.v4.1	1
Pavir.6NG112900.v4.1	-1
Pavir.J000100.v4.1	-1
Pavir.7NG380700.v4.1	-1
Pavir.8KG000800.v4.1	-1
Pavir.9NG544900.v4.1	-1
Pavir.9NG073600.v4.1	1
Pavir.1KG456400.v4.1	1
Pavir.6NG339700.v4.1	1
Pavir.8KG189000.v4.1	-1
Pavir.9KG481700.v4.1	-1
Pavir.J384300.v4.1	1

Pavir.2KG320000.v4.1	-1
Pavir.7KG219100.v4.1	1
Pavir.5KG199300.v4.1	-1
Pavir.2NG060800.v4.1	1
Pavir.6KG283400.v4.1	1
Pavir.4KG352300.v4.1	1
Pavir.6KG359000.v4.1	1
Pavir.6KG359300.v4.1	-1
Pavir.6NG051000.v4.1	-1
Pavir.6KG113500.v4.1	1
Pavir.J727300.v4.1	1
Pavir.7KG075300.v4.1	-1
Pavir.5KG348500.v4.1	-1
Pavir.1NG253000.v4.1	-1
Pavir.9KG498800.v4.1	1
Pavir.J191800.v4.1	-1
Pavir.5KG209900.v4.1	-1
Pavir.7NG058800.v4.1	1
Pavir.3KG315400.v4.1	-1
Pavir.9KG564100.v4.1	1
Pavir.8KG330200.v4.1	-1
Pavir.3NG240800.v4.1	-1
Pavir.J569300.v4.1	-1
Pavir.3KG001400.v4.1	-1
Pavir.7KG095600.v4.1	1
Pavir.2KG178200.v4.1	-1
Pavir.1NG162500.v4.1	-1
Pavir.8NG076100.v4.1	1
Pavir.3KG284800.v4.1	1
Pavir.5KG721300.v4.1	-1
Pavir.2KG334600.v4.1	1
Pavir.3KG217500.v4.1	-1
Pavir.4KG101000.v4.1	-1
Pavir.7NG309200.v4.1	1
Pavir.8NG316700.v4.1	1
Pavir.7KG224100.v4.1	-1
Pavir.6KG111600.v4.1	-1
Pavir.2KG200900.v4.1	-1
Pavir.1KG100500.v4.1	1
Pavir.7NG076500.v4.1	1
Pavir.2NG151800.v4.1	-1
Pavir.4NG037700.v4.1	-1
Pavir.2NG291400.v4.1	-1
Pavir.J397700.v4.1	-1
Pavir.8NG086600.v4.1	-1
Pavir.5KG302800.v4.1	1
Pavir.J779200.v4.1	1
Pavir.2NG003200.v4.1	-1
Pavir.3NG168700.v4.1	-1
Pavir.8KG041400.v4.1	-1
Pavir.3KG056500.v4.1	1
Pavir.8NG042400.v4.1	-1
Pavir.5KG205700.v4.1	-1
Pavir.8KG321500.v4.1	1

Pavir.8NG007200.v4.1	1
Pavir.3NG002700.v4.1	-1
Pavir.2KG292700.v4.1	1
Pavir.J539000.v4.1	1
Pavir.7NG295400.v4.1	-1
Pavir.5KG662600.v4.1	-1
Pavir.1NG082100.v4.1	-1
Pavir.5NG476600.v4.1	-1
Pavir.5KG466500.v4.1	1
Pavir.9NG498900.v4.1	-1
Pavir.5KG302900.v4.1	1
Pavir.4NG139000.v4.1	1

Legend: The gene ID from switchgrass (*Panicum virgatum*) has been extracted. The experiment file has three possible values: 0, 1, and -1. i) "0" means a given gene was not identified as responsive in a particular condition. ii) "1" means the gene was identified as responsive and showed upregulated in at least one of the comparisons in a specific condition. iii) "-1" means the gene was identified as responsive and showed down-regulated in the comparisons.
