# Supplementary material for: Genome-wide profiling of histone (H3) lysine 4 (K4) tri-methylation (me3) under drought, heat, and combined stresses in switchgrass
Source: BMC Genomics. 2024 Feb 29;25:223. doi: 10.1186/s12864-024-10068-w (PMC10903042; doi:10.1186/s12864-024-10068-w)
Supplement: Supplementary file 8 — Additional file 8: Supplemental Table 8. List of DTHT responsive genes overlapping DTHT responsive peaks (for MapMan visualization). [file 12864_2024_10068_MOESM8_ESM.pdf]

Supplemental Table 8. List of DTHT responsive genes overlapping DTHT responsive peaks (for MapMan visualization)

	DTHTvsC
Gene ID	Possible values
Pavir.6KG298400.v4.1	0
Pavir.9KG015400.v4.1	0
Pavir.2NG003200.v4.1	-1
Pavir.9NG733000.v4.1	0
Pavir.6NG051000.v4.1	0
Pavir.8KG330200.v4.1	-1
Pavir.7KG032000.v4.1	1
Pavir.7NG058800.v4.1	0
Pavir.2KG178200.v4.1	-1
Pavir.7NG198300.v4.1	0
Pavir.6KG122600.v4.1	0
Pavir.5NG443900.v4.1	0
Pavir.9KG354500.v4.1	0
Pavir.9NG799200.v4.1	0
Pavir.9KG394200.v4.1	1
Pavir.3KG414500.v4.1	0
Pavir.7NG309200.v4.1	1
Pavir.9NG623800.v4.1	0
Pavir.1KG387700.v4.1	0
Pavir.5KG674200.v4.1	0
Pavir.5NG481800.v4.1	0
Pavir.3KG053600.v4.1	1
Pavir.J397700.v4.1	0
Pavir.5KG205700.v4.1	-1
Pavir.2KG077700.v4.1	-1
Pavir.2NG357300.v4.1	0
Pavir.J263400.v4.1	1
Pavir.8KG242500.v4.1	0
Pavir.8KG173000.v4.1	1
Pavir.4NG101300.v4.1	0
Pavir.9NG161200.v4.1	0
Pavir.8KG189000.v4.1	-1
Pavir.1NG519200.v4.1	1
Pavir.7NG076500.v4.1	0
Pavir.4KG246000.v4.1	0
Pavir.7NG059100.v4.1	0
Pavir.J191800.v4.1	-1
Pavir.4KG101000.v4.1	-1
Pavir.6KG378900.v4.1	-1
Pavir.8NG086600.v4.1	-1
Pavir.6NG211800.v4.1	1
Pavir.5KG674400.v4.1	0
Pavir.3NG043700.v4.1	-1
Pavir.1NG229200.v4.1	0
Pavir.5NG512500.v4.1	1
Pavir.5KG482900.v4.1	-1
Pavir.9KG214700.v4.1	1
Pavir.8NG245500.v4.1	0
Pavir.J015000.v4.1	0
Pavir.3KG272800.v4.1	0
Pavir.9NG855200.v4.1	-1
Pavir.J000100.v4.1	0

Pavir.9KG184300.v4.1	0
Pavir.7KG118800.v4.1	0
Pavir.5KG579400.v4.1	1
Pavir.9KG163500.v4.1	0
Pavir.5KG662600.v4.1	0
Pavir.2KG516400.v4.1	0
Pavir.5KG667500.v4.1	0
Pavir.8NG007200.v4.1	1
Pavir.9NG353300.v4.1	0
Pavir.1KG234200.v4.1	0
Pavir.5KG302900.v4.1	1
Pavir.7KG346000.v4.1	0
Pavir.7NG354300.v4.1	0
Pavir.J416100.v4.1	0
Pavir.2NG198200.v4.1	0
Pavir.8NG197300.v4.1	0
Pavir.8KG001600.v4.1	1
Pavir.3KG056500.v4.1	0
Pavir.8NG001000.v4.1	0
Pavir.7KG075300.v4.1	-1
Pavir.7KG224100.v4.1	0
Pavir.6NG085900.v4.1	-1
Pavir.3KG246800.v4.1	0
Pavir.7KG047300.v4.1	0
Pavir.7NG207500.v4.1	0
Pavir.1KG144100.v4.1	1
Pavir.J193100.v4.1	0
Pavir.4KG300700.v4.1	0
Pavir.2NG060800.v4.1	0
Pavir.4NG036000.v4.1	0
Pavir.9KG121500.v4.1	0
Pavir.5KG302800.v4.1	1
Pavir.5NG536100.v4.1	1
Pavir.3KG332900.v4.1	0
Pavir.1KG097400.v4.1	0
Pavir.J014700.v4.1	0
Pavir.9NG498900.v4.1	0
Pavir.2NG118800.v4.1	1
Pavir.1KG368100.v4.1	0
Pavir.2NG015100.v4.1	-1
Pavir.J569300.v4.1	-1
Pavir.2KG204500.v4.1	0
Pavir.2NG009500.v4.1	0
Pavir.9NG501700.v4.1	0
Pavir.6KG183200.v4.1	1
Pavir.1NG381400.v4.1	0
Pavir.5NG231300.v4.1	-1
Pavir.9KG151000.v4.1	1
Pavir.2NG151800.v4.1	-1
Pavir.5KG678000.v4.1	0
Pavir.5KG305800.v4.1	0
Pavir.5NG381400.v4.1	0
Pavir.3NG175400.v4.1	0
Pavir.8NG058300.v4.1	0

Pavir.2KG528100.v4.1	0
Pavir.7KG140700.v4.1	0
Pavir.7KG093600.v4.1	1
Pavir.3NG218800.v4.1	-1

Legend: The gene ID from switchgrass (*Panicum virgatum*) has been extracted. The experiment file has three possible values: 0, 1, and -1. i) "0" means a given gene was not identified as responsive in a particular condition. ii) "1" means the gene was identified as responsive and showed upregulated in at least one of the comparisons in a specific condition. iii) "-1" means the gene was identified as responsive and showed down-regulated in the comparisons.
