# Supplementary material for: Genome-wide profiling of histone (H3) lysine 4 (K4) tri-methylation (me3) under drought, heat, and combined stresses in switchgrass
Source: BMC Genomics. 2024 Feb 29;25:223. doi: 10.1186/s12864-024-10068-w (PMC10903042; doi:10.1186/s12864-024-10068-w)
Supplement: Supplementary file 9 — Additional file 9: Supplemental Table 9. List of HT responsive genes overlapping HT responsive peaks (for MapMan visualization). [file 12864_2024_10068_MOESM9_ESM.pdf]

Supplemental Table 9. List of HT responsive genes overlapping HT responsive peaks (for MapMan visualization)

	DTHTvsDT
Gene ID	Possible Values
Pavir.9NG498900.v4.1	0
Pavir.9NG006500.v4.1	0
Pavir.9NG082500.v4.1	0
Pavir.6KG378900.v4.1	0
Pavir.1KG487800.v4.1	1
Pavir.1KG144100.v4.1	0
Pavir.7NG058800.v4.1	0
Pavir.1NG381400.v4.1	1
Pavir.3KG332900.v4.1	0
Pavir.1NG508300.v4.1	0
Pavir.6NG183100.v4.1	0
Pavir.3KG172200.v4.1	1
Pavir.2KG204500.v4.1	1
Pavir.5KG348500.v4.1	0
Pavir.9NG811300.v4.1	-1
Pavir.7KG118800.v4.1	0
Pavir.1KG521300.v4.1	0
Pavir.5KG662600.v4.1	0
Pavir.6NG112900.v4.1	-1
Pavir.4KG101000.v4.1	0
Pavir.6NG085900.v4.1	0
Pavir.9KG151300.v4.1	0
Pavir.7NG316600.v4.1	0
Pavir.7KG249500.v4.1	0
Pavir.J099800.v4.1	0
Pavir.J206400.v4.1	-1
Pavir.9KG564100.v4.1	0
Pavir.1KG097400.v4.1	0
Pavir.6NG051000.v4.1	0
Pavir.5NG476600.v4.1	0
Pavir.5KG627200.v4.1	0
Pavir.8NG197300.v4.1	-1
Pavir.8KG189000.v4.1	0
Pavir.2NG198200.v4.1	0
Pavir.6KG359000.v4.1	0
Pavir.4KG402100.v4.1	0
Pavir.5KG302800.v4.1	0
Pavir.9NG073600.v4.1	0
Pavir.2NG561600.v4.1	0
Pavir.2KG178200.v4.1	0
Pavir.9KG151200.v4.1	1
Pavir.9KG151000.v4.1	0
Pavir.6KG165700.v4.1	0
Pavir.3NG027500.v4.1	1
Pavir.8NG042400.v4.1	0
Pavir.3KG217500.v4.1	0
Pavir.7NG380700.v4.1	0
Pavir.3NG240800.v4.1	0
Pavir.7NG295400.v4.1	0
Pavir.8NG352200.v4.1	0
Pavir.3KG284800.v4.1	0
Pavir.9KG191200.v4.1	0
Pavir.7NG021500.v4.1	0
Pavir.7NG043100.v4.1	0
Pavir.2KG334600.v4.1	0
Pavir.7KG260600.v4.1	0
Pavir.6KG113500.v4.1	0
Pavir.2KG292700.v4.1	0
Pavir.J384300.v4.1	0
Pavir.5KG302900.v4.1	0
Pavir.2NG320900.v4.1	0
Pavir.4NG037700.v4.1	1

Pavir.8KG121200.v4.1	0
Pavir.8KG000800.v4.1	0
Pavir.9KG620200.v4.1	-1
Pavir.4NG174500.v4.1	0
Pavir.8KG242500.v4.1	1
Pavir.5KG663800.v4.1	0
Pavir.J191800.v4.1	0
Pavir.9KG518000.v4.1	1
Pavir.8KG321500.v4.1	0
Pavir.3KG195400.v4.1	0
Pavir.J539000.v4.1	0
Pavir.7KG140700.v4.1	0
Pavir.6KG359300.v4.1	0
Pavir.3NG168700.v4.1	0
Pavir.6NG235400.v4.1	0
Pavir.3NG079500.v4.1	0
Pavir.7NG076500.v4.1	0
Pavir.1NG544800.v4.1	0
Pavir.6KG111600.v4.1	0
Pavir.6KG112900.v4.1	0
Pavir.9NG733000.v4.1	0
Pavir.1NG087100.v4.1	0
Pavir.9NG722500.v4.1	0
Pavir.7KG095600.v4.1	0
Pavir.7NG180700.v4.1	0
Pavir.7NG070800.v4.1	0
Pavir.J320300.v4.1	0
Pavir.2NG003200.v4.1	0
Pavir.4NG036000.v4.1	0
Pavir.2NG068500.v4.1	0
Pavir.9KG445800.v4.1	0
Pavir.5KG575800.v4.1	-1
Pavir.7NG362400.v4.1	0
Pavir.8NG086600.v4.1	0
Pavir.4KG352300.v4.1	-1
Pavir.5KG170000.v4.1	0
Pavir.2NG231900.v4.1	0
Pavir.9KG053100.v4.1	0
Pavir.7KG075300.v4.1	0
Pavir.6KG114800.v4.1	0
Pavir.3KG315400.v4.1	0
Pavir.6KG109000.v4.1	0
Pavir.9NG544900.v4.1	0
Pavir.7KG339600.v4.1	0
Pavir.2KG320000.v4.1	0
Pavir.5KG760600.v4.1	0
Pavir.2NG291400.v4.1	0
Pavir.9KG394200.v4.1	0
Pavir.J727300.v4.1	0
Pavir.2NG015100.v4.1	0
Pavir.8NG076100.v4.1	-1
Pavir.2NG118800.v4.1	0
Pavir.5NG254900.v4.1	0
Pavir.J000100.v4.1	0
Pavir.3NG109300.v4.1	0
Pavir.5KG199300.v4.1	0
Pavir.5KG305800.v4.1	0
Pavir.3NG298900.v4.1	0
Pavir.1NG082100.v4.1	0
Pavir.6KG113600.v4.1	0
Pavir.5KG646800.v4.1	0
Pavir.2NG060800.v4.1	0
Pavir.7NG059100.v4.1	0
Pavir.3NG002700.v4.1	0

Pavir.3NG226100.v4.1	0
Pavir.6KG113300.v4.1	0
Pavir.1KG140000.v4.1	0
Pavir.1KG490600.v4.1	0
Pavir.9NG853600.v4.1	0
Pavir.3KG001400.v4.1	0
Pavir.9KG498800.v4.1	-1
Pavir.6KG115200.v4.1	0
Pavir.1KG525800.v4.1	0
Pavir.J015000.v4.1	0

Legend: The gene ID from switchgrass (*Panicum virgatum*) has been extracted. The experiment file has three possible values: 0, 1, and -1. i) "0" means a given gene was not identified as responsive in a particular condition. ii) "1" means the gene was identified as responsive and showed upregulated in at least one of the comparisons in a specific condition. iii) "-1" means the gene was identified as responsive and showed down-regulated in the comparisons.
