# Supplementary material for: Genome-wide profiling of histone (H3) lysine 4 (K4) tri-methylation (me3) under drought, heat, and combined stresses in switchgrass
Source: BMC Genomics. 2024 Feb 29;25:223. doi: 10.1186/s12864-024-10068-w (PMC10903042; doi:10.1186/s12864-024-10068-w)
Supplement: Supplementary file 10 — Additional file 10: Supplemental Figure 1. Distribution of stress-responsive peaks based on genomic features. [file 12864_2024_10068_MOESM10_ESM.pdf]

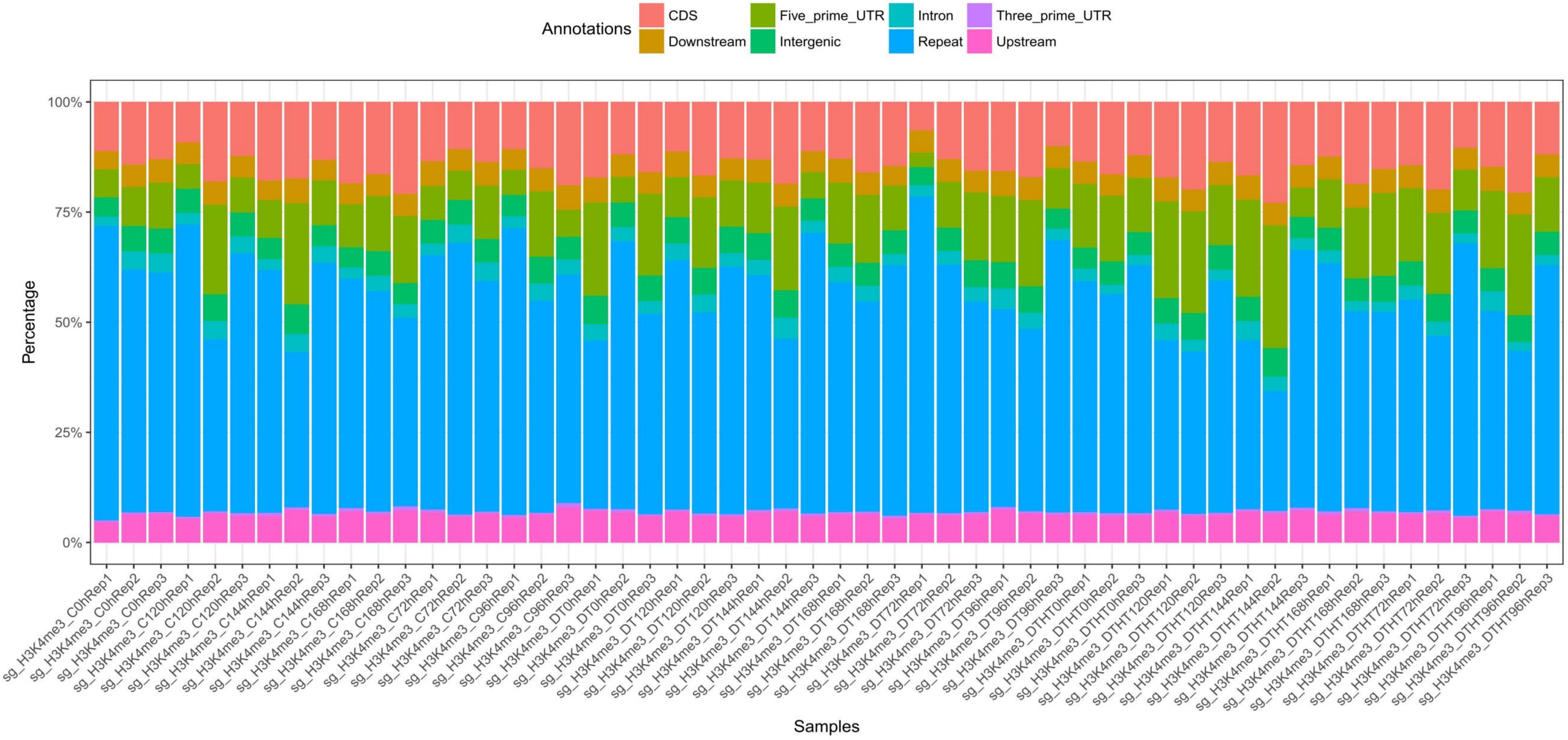

Supplemental Figure 1. Distribution of stress-responsive peaks based on genomic features. X-axis represents the H3K4me3 marked Control, Drought, and Combined Drought and Heat samples of Switchgrass presented as three replicates per each collection timepoint (as per the experimental design discussed in methods). Y-axis represents the %peaks identified per genomic feature (upstream, 3' UTR, repeat, intron, intergenic, 5' UTR, downstream, and CDS regions) in Switchgrass.
